# Epidemiological and virological surveillance of the prevention of mother-to-child transmission of HIV among pregnant women in Togo

**DOI:** 10.1186/s12884-024-06435-w

**Published:** 2024-04-15

**Authors:** Kokou Tegueni, Fifonsi Adjidossi Gbeasor-Komlanvi, Oumarou I. Wone Adama, Arnold Junior Sadio, Amivi Phyllis Amenyah-Ehlan, Claver Anoumou Dagnra, Didier Koumavi Ekouevi

**Affiliations:** 1 Département des Sciences Fondamentales, Laboratoire de Biologie Moléculaire et d’Immunologie (BIOLIM- FSS/UL), Université de Lomé, Lomé, Togo; 2 Programme National de Lutte contre le VIH/Sida, les Hépatites virales et les Infections Sexuellement Transmissibles (PNLS/HV/IST), Lomé, Togo; 3 Laboratoire National de Référence pour tests VIH, les Hépatites virales et les Infections Sexuellement Transmissibles (LNR/HV/IST), Lomé, Togo; 4 Département de Santé Publique, Faculté des Sciences de la Santé, Université de Lomé, Lomé, Togo; 5 Centre Africain de Recherche en Epidémiologie et en Santé Publique (CARESP), Lomé, Togo; 6 Global Health in the Global South (GHiGS) Team, Bordeaux Population Health Research Center, University of Bordeaux, National Institute for Health and Medical Research (INSERM) UMR 1219, Research Institute for Sustainable Development (IRD) EMR 271, Bordeaux, France; 7 Institut de Santé Publique Epidémiologie Développement (ISPED), Université de Bordeaux, Bordeaux, France

**Keywords:** Mother-to child transmission, HIV, Viral load, Togo

## Abstract

**Background:**

In 2015, Togo introduced the “test-and-treat” strategy for the prevention of mother-to-child transmission (PMTCT) of HIV. Pediatric HIV infection remains a public health problem in Togo, with a mother-to-child transmission (MTCT) rate of 3.6% in 2020. This study aimed to estimate cases of HIV seroconversion during pregnancy and to identify pregnant women at high risk of transmitting HIV to their children in Lomé, Togo.

**Methods:**

A descriptive cross-sectional study was carried out from 18 March to 22 May 2022 among women who had given birth in five maternity units providing PMTCT services in Lomé. Umbilical cord blood samples were taken from the maternal side by midwives after delivery. HIV serology was performed in the laboratory using the Alere™ HIV Combo SET and First Response HIV 1–2. Card Test version 2.0. A sample was considered positive if both tests were positive. The HIV-1 viral load in HIV-1-positive samples was measured using Cobas/Roche 4800 equipment. Information on the women was extracted from maternal antenatal records and antenatal consultation registers.

**Results:**

A total of 3148 umbilical cord blood samples (median maternal age: 28 years (interquartile range [24–32]) were collected. Among them, 99.3% (3145/3148) had presented for at least one antenatal clinic visit before giving birth, and 78.7% (2456/3122) had presented for at least four visits. One hundred and twenty-one (121) cord samples were HIV-1 positive, representing a seroprevalence of 3.8% (95% CI = [3.2–4.6]). Among them, 67.8% (82/121) were known HIV-positive before the current pregnancy, 29.7 (36/121) were diagnosed as HIV-positive at the antenatal visits and 2.5% (3/121) were diagnosed as HIV-positive in the delivery room. Of the HIV-positive women, 85.9% (104/121) were on ARV treatment before delivery. The viral load was < 1000 copies/ml in 97.5% (118/121) cases.

**Conclusion:**

This study explored the virologic and epidemiological aspects of HIV among pregnant women in Togo. The results show significant viral suppression at delivery in women ART. Surveillance based on umbilical cord blood specimen screening is an interesting approach for monitoring the effectiveness of PMTCT programmes.

## Background

According to the Joint United Nations Program on HIV/AIDS (UNAIDS) estimates for 2021, the pediatric HIV epidemic remains a public health problem, with 1.7 million children aged 0–14 years living with HIV and 150,000 children newly infected worldwide in 2021 [1]. Worldwide, mother-to-child transmission (MTCT) of HIV accounts for more than 90% of all new pediatric HIV infections [2]. In 2020, 89% of new pediatric HIV infections and 88% of HIV-positive children and adolescents worldwide were reported in sub-Saharan Africa [3].

Vertical transmission of HIV can occur during pregnancy, labor, delivery or breastfeeding. Antiretroviral treatment (ART), as part of the prevention of mother-to-child transmission (PMTCT), is a key strategy for combating the HIV epidemic and has reduced the vertical transmission rate from 45% to less than 5% in breastfeeding populations [4]. Early initiation of antiretroviral treatment in the first trimester of pregnancy reduces the transmission rate to less than 1% [5].

PMTCT of HIV, which is an important part of the overall management of HIV/AIDS infection, remains a challenge in most resource-limited countries, particularly in Africa [6]. In 2009, the UNAIDS called for the virtual elimination of MTCT for the first time, aiming to reduce the vertical transmission rate to less than 5% in breastfeeding women and to 2% or less in no breastfeeding women [7]. However, there are few data on the elimination of pediatric HIV.

In Togo, according to UNAIDS estimates, HIV prevalence was 1.9% in the general population aged 15–49 years in 2021 [8]. Regarding PMTCT, since 2012, significant progress has been made with the introduction and widespread use of triple antiretroviral therapy in all PMTCT sites, particularly the delegation of ART initiation and follow-up of HIV-infected pregnant women by PMTCT site providers (midwives, nurses, birth attendants) [9]. In 2021, 96% of pregnant women were receiving ART, and 53% of children born to HIV-positive mothers were receiving ARV prophylaxis; however, the rate of MTCT was estimated at 2.9% [10]. There are few data on viral suppression at the time of delivery and on adherence to antiretroviral treatment. In the absence of a second HIV test during pregnancy, data on seroconversion are very limited [11]. The aim of this study was to estimate cases of HIV seroconversion during pregnancy and to identify pregnant women at high risk of transmitting HIV to their child in 5 PMTCT centers in Lomé, Togo, in 2022.

## Methods

### Study design and period

We conducted an anonymous, uncorrelated cross-sectional study from 18 March to 22 May 2022 in five maternity units in Lomé, Togo. The study consisted of taking umbilical cord blood samples from the maternal side after delivery for all women admitted to the health centers selected for the study. This was therefore a modified uncorrelated screening. An approach already used in other studies and usually coupled with pharmacological assays [12–14].

### Study setting and population

The study was carried out in the maternity wards of five health centers in Lomé, the economic and political capital of Togo. These were the *CHU Sylvanus Olympio, Hôpital de Bè*, *Centre Médicosocial* (CMS) *Cacaveli*, CMS *Adidogome*, and *Association Togolaise pour le Bien-Être Familial- Lomé*. These centers offer all PMTCT services and were selected on the basis of the average number of deliveries per month (≥ 50 deliveries/month). The PMTCT services in Togo include an antenatal HIV screening, a treatment initiation the same day as diagnosis with tenofovir, lamivudine and dolutegravir. Newborns receive nevirapine with maternal breastfeeding for at least six months.

The study population consisted of women admitted for childbirth. The sample size calculation was based on several studies which report HIV seroconversion among pregnant women in Cameroon and south Africa. In these studies the prevalence of HIV seroconversion among pregnant women was around 2.0-7.9% [15, 16].

We hypothesized that seroconversion in Togo would be much lower, around 1%, based on the following factors: (i) HIV prevalence in Togo is much lower than in these two countries [8, 17, 18]; (ii) the seroconversion data reported here for these countries are several years old and should now be lower than those reported.

Based on this hypothesis of a 1% seroconversion rate for Togo, with a margin of error of 0.3%, and an expected non-response rate of 5%, with 95% confidence, the estimated minimum sample size was 3079.

Women with stillbirths and macerated stillbirths were not included in this study because of blood coagulation. Women with unhealthy and broken umbilical cords, those referred to the maternity unit surveyed before delivery, and those who did not deliver in the center were the criteria for non-inclusion in our study.

### Data collection

Data were collected for women who presented to the centers for delivery by midwives using the antenatal consultation booklet. The data collected covered the sociodemographic characteristics, gynecological and obstetric history, HIV testing history, and management of HIV-infected women. We also collected information from maternal antenatal records and antenatal consultation registers for pregnant women. This information was recorded on standardized anonymous forms and linked to the cord blood sample using a unique identification number. The data collected from each maternal prenatal record and prenatal consultation register were as follows: maternal age, gestational age, parity, number of prenatal visits, place of the first prenatal visit, proposal and acceptance of HIV screening before and during pregnancy, HIV serology, ARV treatment received, administration of nevirapine to the child at birth, time of administration of nevirapine after birth, initiation of replacement feeding in the newborn.

### Biological aspects

In each hospital, midwives took anonymous samples of blood from the maternal side of the umbilical cord using a 10 ml syringe. Ten milliliters of whole blood were collected from each woman after delivery of the placenta and divided into two EDTA tubes bearing a unique identification number. The samples were first kept at + 4 °C in the refrigerator at the maternity hospital or at the health center laboratory and then transported to the Molecular Biology and Immunology Laboratory of the Faculty of Health Sciences of the University of Lomé - Togo (BIOLIM/FSS-UL), where all analyses were performed. All samples were centrifuged in the laboratory at 1,500 rpm for 10 min prior to testing.

We used two rapid tests to determine HIV serology. The first test was the AlereTM HIV Combo SET test (Alere Medical Co., Ltd, Chiba, 270–2214, Japan), which is a fourth-generation qualitative immunoassay with 100% sensitivity used for the detection of anti-HIV-1 and anti-HIV-2 antibodies (Abs) and the detection of free, nonimmunocomplexed HIV-1 antigens (Ags) in capillary or venous whole blood, plasma or human serum. The second rapid test used to confirm and distinguish between the two types of HIV was the First Response HIV 1–2. Card Test version 2.0 (A1-302, GIDC, Sarigam 396,155. Dist. Valsad, Gujarat, INDIA). This is an immunochromatographic test with 100% specificity for the qualitative detection of antibodies specific to HIV-1 and HIV-2. A woman was considered to be HIV-infected when both tests were positive.

HIV-1 viral load was determined by real-time polymerase chain reaction (PCR) on all plasma samples with positive HIV-1 serology using Cobas 4800/Roche equipment (Roche Diagnostics, Indianapolis, USA) consisting of the Cobas x480 extractor and the Cobas z 480 analyzer. The viral load detectability threshold of the Cobas 4800 was 50 copies/mL.

We defined as to be at high risk of transmitting HIV to their child, any HIV infected women with HIV-1 viral load above 1000 copies/mL.

### Statistical analysis

Completed questionnaires were checked for completeness and consistency before data were entered. The data were then entered using Epidata 3.1 software. Descriptive statistics were carried out, and the results are presented in tables showing numbers and proportions for the qualitative variables. Quantitative variables are presented as medians with their interquartile ranges (IQRs). The prevalence of HIV-1 in the mothers was estimated with its 95% confidence interval (95% CI). Qualitative variables were compared using the chi-square and/or Fisher test, and the Wilcoxon test was used to compare quantitative variables (medians). All analyses were performed using R version 4.1.3 statistical software.

### Ethical considerations

Our study protocol was approved by the National Bioethics Committee for Health Research - CBRS, (Number°015/2022/CBRS), and we obtained the authorization of all managers from the health centers selected for the study. The CBRS is a governmental agency that validates protocols on an ethical level but remains independent of the research team.

## Results

### Sociodemographic and clinical characteristics of the mother-child pairs

**Fig. 1:**
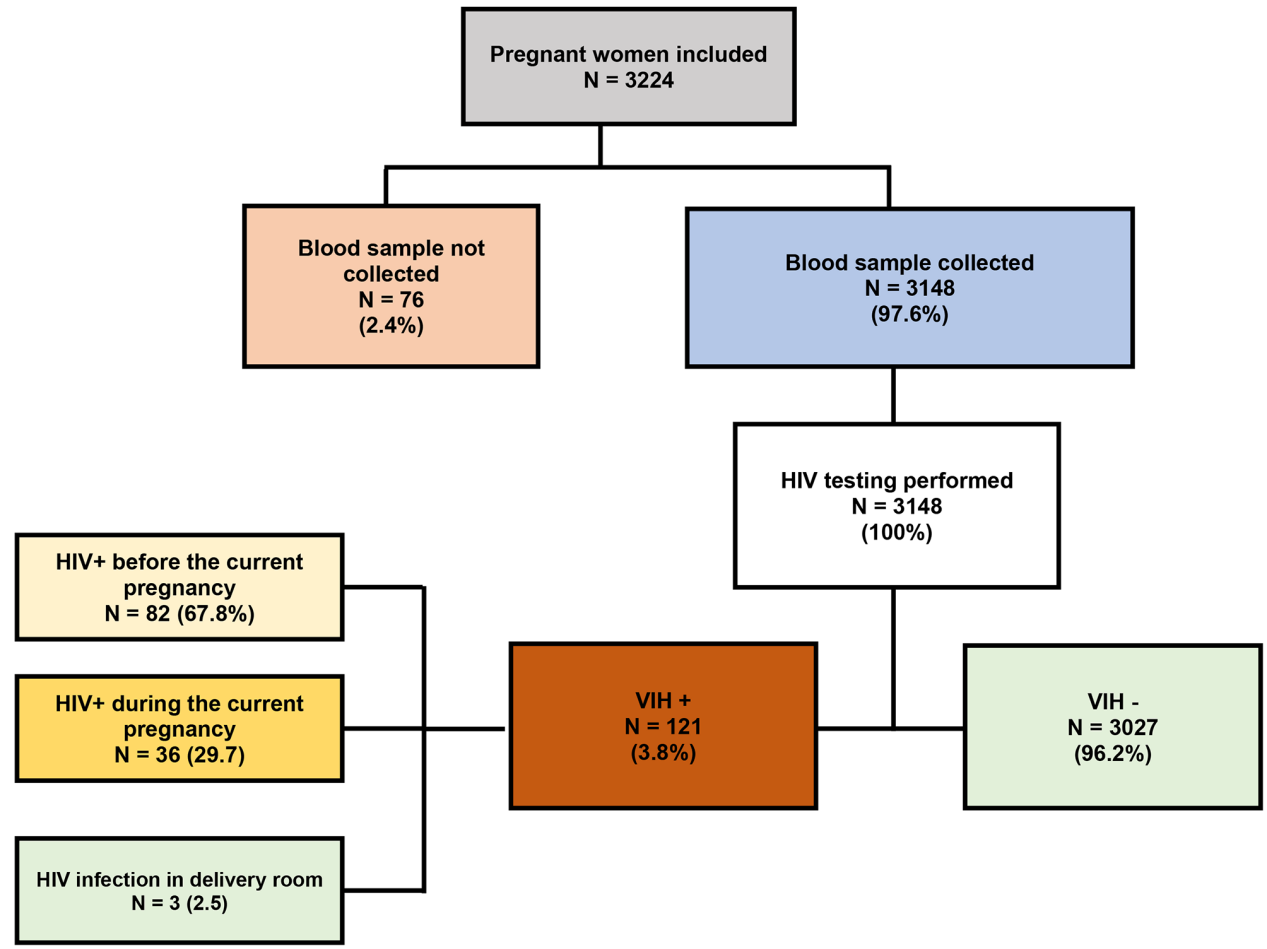
Flow chart of pregnant women included in the study

Between March and May 2022, 3,224 women gave birth at the study sites. A total of 3,148 umbilical cord blood specimens were collected (Fig. 1). The median age of the mothers was 28 years (IIQ [24–32]). Nearly eight out of ten women (78.7%) had presented more than four antenatal clinic visits prior to delivery. More than half of the women had given birth vaginally (55.3%), and 11.0% had babies with low birth weight (< 2500 g; Table 1).

**Table 1:** Socio-demographic characteristics of mother-child pairs according to HIV-1 status based on cord blood testing (*N* = 3 148)

Characteristics	HIV cord blood results			*P*
	Negative	Positive	Total	
	*N* = 3 027	*N* = 121	*N* = 3 148	
**Age range (years). n (%)**				**< 0.001** ^*1*^
< 25	858 (98.7)	11 (1.3)	869	
25–34	1 728 (96.3)	67 (3.7)	1 795	
35+	441 (91.1)	43 (8.9)	484	
**Gestity. n (%)**				**< 0.001** ^*1*^
1–2	1 755 (97.5)	45 (2.5)	1 800	
3–4	934 (94.2)	57 (5.8)	991	
5+	330 (94.6)	19 (5.4)	349	
Missing	08		08	
**Parity. n (%)**				0.904^*2*^
1–2	2 415 (96.1)	99 (3.9)	2 514	
3–4	492 (96.3)	19 (3.7)	511	
5+	107 (97.3)	3 (2.7)	110	
Missing	13		13	
**Number of ANC visit. n (%)**				0.331^*2*^
0	21 (91.3)	2 (8.7)	23	
< 4	616 (95.8)	27 (4.2)	643	
4+	2 364 (96.3)	92 (3.7)	2 456	
Missing	26		26	
**Mode of delivery. n (%)**				> 0.999^*2*^
Low track	1 672 (96.2)	67 (3.8)	1 739	
Instrumental	3 (100.0)	0 (0.0)	3	
Caesarean section	1 352 (96.2)	54 (3.8)	1 406	
**Birth weight (g). n (%)**				0.061^*1*^
Low weight (< 2500)	328 (94.5)	19 (5.7)	347	
Normal weight (2500+)	2 699 (96.4)	102 (3.6)	2 801	

ANC = antenatal consultation; p = value of p

### History of maternal HIV testing and the prevalence of HIV in cord blood

Regarding the maternal history of HIV testing, based on the data collected from the ANC booklet, 950 (30.2%) women had already been screened before the current pregnancy. During the current pregnancy, 3030 pregnant women (96.3%) received pretest counseling and underwent an HIV test. Among them, 118 women were tested positive, giving an HIV-1 prevalence of 3.9% (95% CI [3.2–4.7]).

A total of 3148 cord blood specimens were collected and tested for HIV (Figs. 1), 121 were positive for HIV-1, giving a seroprevalence of 3.8% (95% CI [3.2–4.6]) (Table 2). Among them, 67.8% (82/121) were known HIV positive before the current pregnancy, 29.7% (36/121) were diagnosed as HIV-positive at the antenatal visits during the current pregnancy and 2.5% (3/121) were diagnosed as HIV-positive in the delivery room (Fig. 1).

**Table 2:** History of HIV testing of sampled women who have given birth (*N* = 3148)

Characteristics	Number	Proportion (%)
**HIV testing before the current pregnancy**		
No	1538	48.9
Yes	950	30.2
Don’t know	660	20.9
**Result of HIV testing before the current pregnancy **(*n*** = 950) **		
Negative	823	86.6
Positive	80	8.4
No information	47	4.9
**HIV screening during current pregnancy**		
No	118	3.7
Yes	3030	96.3
**Results of HIV screening during current pregnancy **(*N*** = 3030)**		
Negative	2893	95.5
Positive	118	3.9
No information	19	0.6
**Results of HIV screening of umbilical cord blood**		
Negative	3 027	96.2
Positive	121	3.8

### Crosstabulation of HIV test results in the cord blood specimens and in the ANC booklet

Table 3 summarizes the HIV test result in the cord blood specimens and in the ANC booklet. Three women were tested positive among 2893 women classified negative based on ANC booklet. Thus, the proportion of HIV seroconversion was 1.0 per 1000 (3/2893) women.

**Table 3:** Crosstabulation of HIV test result in the cord blood and in the ANC booklet

		Cord blood testing, n(%)	
		HIV+ *N* = 121 n (%)	HIV- *n* = 3027 n (%)
ANC booklet test reported, n (%)	HIV+ (*n* = 118)	118 (100,0)	0 (0,0)
	HIV- (*n* = 2893)	3 (0,1)	2890 (99,9)
	Result not available (*n* = 19)	0 (0,00)	19 (100,0)
	HIV test not performed (*n* = 118)	0 (0,00)	118 (100,0)

Row percentage was reported

### Antiretroviral treatment in HIV-1-infected women

The proportion of women who were on ARVs before delivery was 104/121 (85.9%). The ART treatment status was not known for 9.2% (11/121) and 6 were not on treatment (Table 4). The HIV viral load was below 50 copies/mL in 91.7% (111/121) of HIV infected women in the delivery room. This proportion was 95.2% (99/104) among women on treatment and 33.3% (2/6) among women who was not on treatment (Table 4). Only three women (2.5%) was considered as at high risk of HIV transmission to their child (HIV VL ≥ 1000 copies/mL).

**Table 4:** Virological suppression of HIV-positive pregnant women according to ARV treatment initiated (*N* = 121)

	HIV-1 viral load (copies/mL)			Total	
ARV treatment for the mother. n (%)	**< 50** (*n* = 111)	**50–999** (*n* = 7)	**≥ 1000** (*n* = 3)		
No	2 (33.3)	1 (16.7)	3 (50.0)	6 (4.9)	
Yes^$^	99 (95.2)	5 (4.8)	0 (0.0)	104 (85.9)	
Don’t know	10 (90.9)	1 (9.1)	0 (0.0)	11(9.2)	

^$^All women were on DTG-based regimen (Tenofovir/Lamivudine/Dolutegravir)

### PMTCT cascade indicator

**Fig. 2:**
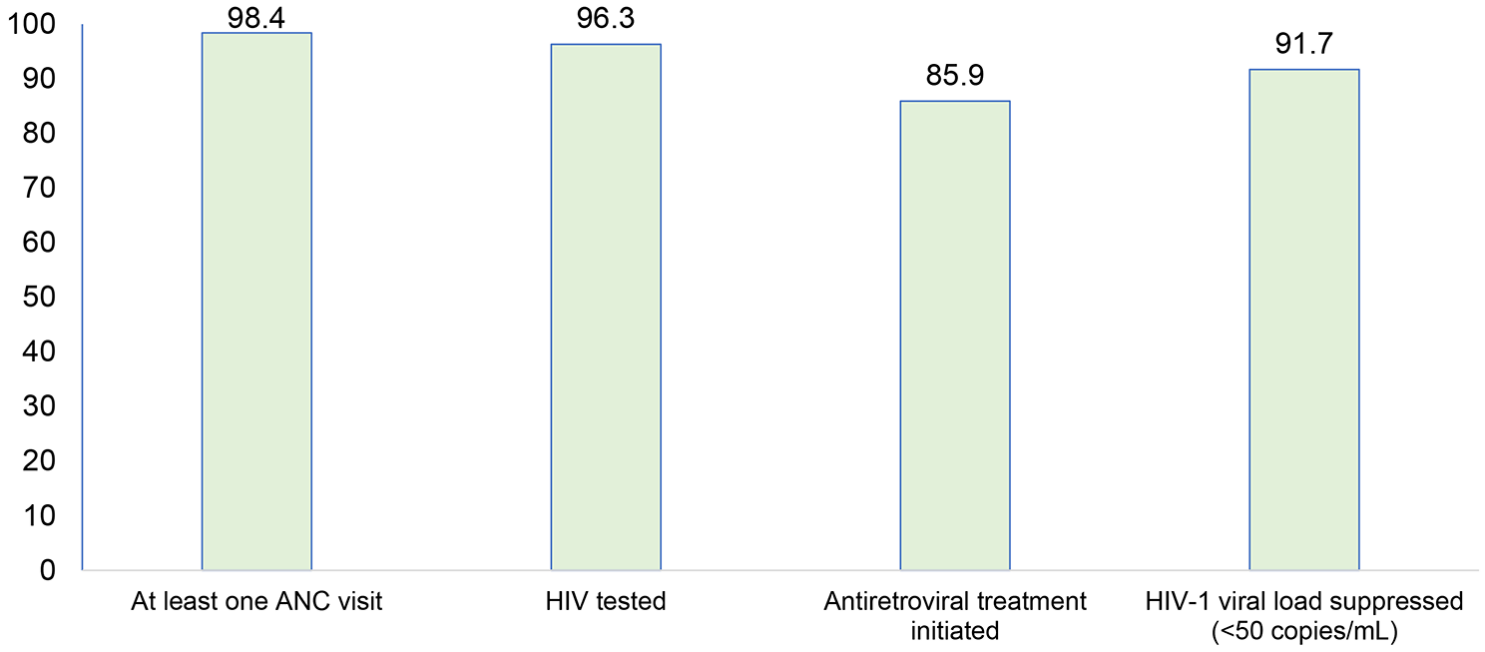
PMTCT cascade indicators

Figure 2 presents the PMTCT cascade indicator from ANC visits to HIV virologic suppression. A total of 98.4% of women performed at least one ANC visit, and 91.7% had HIV-1 virologic suppression.

## Discussion

In this study, we documented cases of HIV seroconversion in pregnant women and identified those at high risk of MTCT. This anonymous, HIV surveillance study using umbilical cord blood specimens reported an HIV prevalence of 3.8% and 1 new HIV case per 1000 tests performed. In addition, more than nine out of ten women (91.7%) had a suppressed viral load at the time of delivery.

Improving pregnancy surveillance is a prioritized public health objective worldwide, particularly in developing countries [19]. The WHO recommends that ANC should be initiated during the first trimester of pregnancy with at least four focused ANC visits and, ideally, eight ANC visits [20]. In regions where maternal mortality is high, such as sub-Saharan Africa, the figures are not encouraging, with only approximately half of pregnant women (49%) realized at least four antenatal visits [21]. In our study, 98.4% of women underwent at least one ANC visit prior to delivery, and nearly eight out of ten (78.7%) underwent at least four. This high rate of use of ANC services may be due to fact that the study has been carried out in capital city of Lomé were the access to health services is more easer.

In our study, 96.3% of women had been tested for HIV according to the data in the ANC booklet. In the city of Lomé, the first 95 UNAIDS targets for HIV testing among pregnant women are reached. This high proportion could be explained by the fact that HIV screening during pregnancy is systematically offered to women.

HIV prevalence based on ANC booklet did not differ from HIV prevalence based on umbilical cord blood specimens (3.9% vs. 3.8%). In addition, all women identified as HIV-positive on the ANC booklet were confirmed positive on the umbilical cord blood specimens, reflecting the reliability of the data in the ANC booklet. In this study, 23 women had no antenatal visits and 649 had fewer than 4 antenatal visits. Non-attendance at antenatal clinics may influence HIV prevalence, as women who do not attend ANC are at greater risk of HIV infection.

The WHO recommends repeat HIV testing for pregnant and postpartum women in countries with a generalized HIV epidemic to identify women with incident infections occurring during pregnancy and breastfeeding [22]. This recommendation is not performed in routine for many reasons (cost, overload of staff). Our survey shows 3 new cases of HIV infection among 2893 women. To avoid missed opportunities for diagnose and treatment, and to reduce risk of MTCT by breastfeeding, this strategy should be implemented specifically in settings with a generalized HIV epidemic.

The number of new cases detected in relation to the number of tests performed seems low, and it should be emphasized that the decision to carry out a second screening test on pregnant women will have to be based on an overall assessment of other factors such as the resources available and national and international guidelines on HIV screening during pregnancy. Cost-effectiveness studies could also provide important information to guide these decisions but should be considered in a much broader context based on the country’s health care needs and resources.

This study also shows that there is an improvement of PMTCT program in Lomé, comparing with the result of the results of a study carried out in the gynecology department of the Sylvanus Olympio University Hospital in Togo in 2010 which showed a discrepancy between the proportion of women diagnosed with HIV infection during ANC visits and the proportion diagnosed in the delivery room [11]. Among the 41 women diagnosed as living with HIV during labor, 34% had not been tested for HIV during pregnancy and were missed opportunities. A decade after this study, we note that almost all women are tested for HIV during prenatal consultations.

The major risk factor of MTCT is the maternal viral load. However, this measurement is not performed in routine to identified women at high risk of transmission. Data on viral load in pregnant and breastfeeding women in sub-Saharan Africa are limited. This study is one of the first to show that viral load is suppressed at delivery in more than 90% of women initiating a dolutegravir-based regimen. The IMPAACT-VESTED trial demonstrated the clear superiority of dolutegravir-based regimens compared with efavirenz in pregnant women. Indeed, at delivery, 98% (398/405) of pregnant women had a suppressed viral load compared to 91% (191/200) of women on efavirenz [23]. This result may also be explained by the fact that 62% of pregnant women had initiated dolutegravir-based regimens before pregnancy. These results support early detection and initiation of dolutegravir-based regimens to achieve viral load suppression before delivery as recommended by WHO. In addition this could also be due to the use of the dolutegravir-based combination, which accelerates viral suppression a few weeks after initiation [24].

To our knowledge, this study is the first of its kind to combine viral load screening and retesting for pregnant women in Lomé. Repeated HIV screening during pregnancy makes it possible to identify women with incident infections or living with HIV who have been lost to follow-up and infants at high risk of HIV infection. This study showed that in Lomé, based on UNAIDS’ 3 × 95, 96.3% of women were screened for HIV during ANC visits, and 91.7% had a suppressed viral load at delivery. These results demonstrate the effectiveness of the PMTCT program in *Lomé*.

This study has some limitations. The first concerns the reliability of the data available in the ANC booklet. However, we noted a good concordance of HIV test results. The second limitation is the absence of pharmacological measurements, particularly in women who were classified as not having initiated treatment or whose ART status was unknown, but who had a suppressed viral load. Also, due to the cross-sectional nature of the study, there was no follow-up, and the rate of mother-to-child transmission was not studied. In addition, the method used in this study, based on maternal umbilical cord blood sampling, does not allow diagnosis of in utero HIV transmission (which accounts for 10 to 20% of transmissions) [25]. Diagnosis after 6 weeks is most important because the majority of MTCT occurs during delivery (40%) or breastfeeding (40%) [25]. Nevertheless, overall, the indicators reported allow us to assess the performance of the PMTCT program in Togo.

## Conclusion

This study explored the virologic and epidemiological aspects of HIV among pregnant women in Togo. Significance progress have been made in PMCT programmes over the last decade. The results show significant viral suppression at delivery in women on ART. Surveillance based on umbilical cord blood specimens screening is an interesting approach for monitoring the effectiveness of PMTCT programmes.

## Data Availability

The datasets used and or analysis during the current study are available from the corresponding author (didier.ekouevi@gmail.com) on reasonable request.

## Abbreviations

ANC: Antenatal care
ART: Antiretroviral therapy
ARV: Antiretroviral
IQR: Interquartile Range
MTCT: Mother-to Child Transmission of HIV
PMTCT: Prevention of Mother-to Child Transmission of HIV
UNAIDS: The Joint United Nations Programme on HIV/AIDS
VL: Viral load
95%CI: 95% Confidence Interval
